# Supplementary material for: Patterns of Intron Gain and Loss in Fungi
Source: PLoS Biol. 2004 Nov 30;2(12):e422. doi: 10.1371/journal.pbio.0020422 (PMC532390; doi:10.1371/journal.pbio.0020422)
Supplement: Table S1 — Also available at http://genes.mit.edu/NielsenEtAl/. (4.3 MB ZIP). [file pbio.0020422.st001.zip › NielsenEtAl/html/572.html]

AN0641.1.NCU06464.1.MG06249.1.FG02523.1


```
 CLUSTAL W (1.82) Multiple Sequence Alignments - Introns Inserted


Sequence 1: NCU06464.1	170 aa
Sequence 2: FG02523.1	170 aa
Sequence 3: MG06249.1	177 aa
Sequence 4: AN0641.1	179 aa
Alignment Length: 185 aa
Number Identitical Residues: 51 aa
Alignment Score (without introns) 3247


MG06249.1 	MLIFK0DIVSGNEMFSDVYDPKLVDDVVYEVDCKMITVDAVNVGA2DATVPD~TGANASA
NCU06464.1	MLIYS0DIITGDEIISDSYDLKEVDGIAYEVDCAMIEEGAVQV--~-----D~IGANASA
FG02523.1 	MLIYN0DILNGDELISDSYDLKEVDGIVYEADCAMIEEGAVEV--~-----N1IGANASA
AN0641.1  	MIIYK0DIISGDEVLSDTYNIKTVDGVLYECDCRKYLKKTNED--~---FEL~EGANPSA
          	*:*:. **:.*:*::** *: * **.: ** **      : :            ***.**

MG06249.1 	EDAEEA--LEDGPTKVNDIIYSFRLQSSSFD------KKSYLSHLKG1FFKAVKEHKKSQ
NCU06464.1	EEADEA--LDDGVVKVNNVVHSFRLQSTQFD------KKGYLVYLKG1YMKSVKNALKEQ
FG02523.1 	EEAAED--LDDGAVKVNNIVNSFRLQSTTFD------KKSYLAYLKG1YMKAIKAKLQEN
AN0641.1  	EGGDDEGGAEGGEVMVHDIEDQFRLVWLKAEDGAKPPKDAFKGHLKT1YMKKVLKALQDK
          	* . : .. :.* . *:::  .***     :..:...*..:  :**  ::* :    :.:

MG06249.1 	GASEEDVKAWEKKAGDYVKNKLLPNFKDFEFYIGEEFNPDGM2VAL0LNYRED0GITPFF
NCU06464.1	GKSDEEIKDFETKASAFAKNVILAKFKDWEFYTGESMNPDGM~VVL~LNYRED~GTTPYV
FG02523.1 	GSSAEDIKAFETGASKFVKDTIVPKFKDFEFYTGESMDPDGM2VVL~LNYRED~GVTPYT
AN0641.1  	GAPDETINEFKKGAPAAVN-KILKNYDNYDVLMGESMDGDAM2HIL~IDFRED~GVTPYA
          	* . * :: ::. *   .:  :: ::.:::.  **.:: *.*   * :::*** * **: 

MG06249.1 	TFWKDGLTEMKC
NCU06464.1	VVWKHGLKETKV
FG02523.1 	IFWKHGLKETKV
AN0641.1  	TLWKHGLEEMKV
          	 .**.** * *
```
